# Low Vitamin D Status Predicts Poor Clinical Outcome in Advanced Melanoma Treated With Immune Checkpoint or BRAF/MEK Inhibitors: A Prospective Non-Interventional Side-by-Side Analysis

**DOI:** 10.3389/fonc.2022.839816

**Published:** 2022-05-20

**Authors:** Jörg Reichrath, Florian Biersack, Stefan Wagenpfeil, Jakob Schöpe, Claudia Pföhler, Roman Saternus, Thomas Vogt

**Affiliations:** ^1^ Department of Dermatology, Saarland University Medical Center, Homburg, Germany; ^2^ Institute of Medical Biometry, Epidemiology and Medical Informatics, Saarland University Medical Center, Homburg, Germany

**Keywords:** vitamin D, vitamin D status, low vitamin D status, advanced melanoma, melanoma, immune checkpoint inhibitor, BRAF/MEK inhibitor

## Abstract

In melanoma and other malignancies, low vitamin D status is associated with increased risk and poor prognosis. However, there are limited data of the impact of 25(OH)D serum concentration (s.c.) on clinical outcome in advanced melanoma. We tested the hypothesis that vitamin D status is predictive of efficacy and safety in patients treated for metastasized melanoma with B-rapidly accelerated fibrosarcoma (BRAF), mitogen-activated protein kinase kinase (MEK), cytotoxic T lymphocyte-associated protein-4 (CTLA-4), and/or programmed cell death protein-1 (PD-1) inhibitors. Severe vitamin D deficiency [defined as 25(OH)D s.c. <10 ng/ml] was associated with markedly reduced overall (OS) and progress-free (PFS) survival, with increased tumor load [TL; measured as s.c. of S100 protein or lactate dehydrogenase (LDH)], and with a trend for higher frequency of adverse events (AEs). An increase in average 25(OH)D s.c. of 1 ng/ml was associated with a 3.9% reduced risk for progressive disease [hazard ratio (HR) = 0.961, *p* = 0.044], with a reduction of LDH s.c. of 3.86 U/l (*p* = 0.034, indicating a reduction of TL), and with a trend for reduced frequency of AEs (AE ratio -0.005; *p* = 0.295). Patients with average 25(OH)D s.c. ≥10 ng/ml and *BRAF*-mutant melanoma showed a trend for a higher frequency of AEs as compared to individuals with *BRAF* wild-type melanomas. Our data indicate that vitamin D deficiency is associated with poor clinical outcome in patients treated for metastasized melanoma with BRAF/MEK inhibitors or immunotherapy. Although it needs to be proven in future interventional trials whether optimizing serum 25(OH)D improves clinical outcome in these patients, we recommend that 25(OH)D s.c. should be analyzed and vitamin D deficiency treated in all patients with advanced melanoma.

## Introduction

Enormous scientific progress in molecular tumor biology and immune surveillance has resulted in the introduction of effective targeted therapies [e.g., by blocking the hyperactive B-rapidly accelerated fibrosarcoma (BRAF)‐mitogen-activated protein kinase (MEK) pathway that represents a hallmark of melanoma] and immunotherapies [including single or dual blockade of cytotoxic T lymphocyte-associated protein-4 (CTLA‐4) and programmed cell death protein-1 (PD-1)] for metastasized melanoma (1–4). An increasing number of clinical trials have impressively shown that BRAF inhibitors (BRAFi; such as dabrafenib, vemurafenib, and encorafenib), MEK inhibitors (MEKi; such as trametinib, cobimetinib, and binimetinib), and immune checkpoint inhibitors [ICIs; including CTLA-4 inhibitors (CTLA-4i) and PD-1 inhibitors (PD-1i), such as ipilimumab and pembrolizumab, nivolumab, respectively] improve clinical outcomes when compared to chemotherapy (1, 3). The relevance of the immune system in controlling melanoma is generally accepted, and since the introduction of CTLA-4 single blockage (ipilimumab) in 2011, clinical application of ICIs for melanoma treatment has shown for the first time in history the impressive therapeutic potential of drugs that regulate immunosurveillance (1, 3). Systemic treatment of advanced melanoma has moved forward rapidly, and although these new treatment modalities have completely changed the reality from one of poor responses and short survival to a very promising new clinical picture of impressively high response rates, greatly prolonged disease control, and the possibility of aiming at the ultimate goal of a cure for some patients (1, 3), treatment of advanced melanoma is still challenging.

Consequently, there is urgent need to characterize the molecular mechanisms that exert the therapeutic efficacy of these new treatment modalities and to identify factors that show promise to further optimize melanoma treatment. It has been shown that low levels of vitamin D are associated with poorer prognosis, thicker tumors, ulceration, and increased inflammation in primary melanomas (5, 6). Several independent lines of investigation have supported the hypothesis that 25(OH)D serum concentration (s.c.) may be associated with the risk and prognosis of melanoma. We reported in 2009 in a pilot study (7) significantly reduced 25(OH)D s.c. in stage IV (median 13.10 ng/ml, n = 115) as compared to stage I (median 16.40 ng/ml, n = 50) melanoma patients (*p* = 0.006). In that investigation, a trend toward a greater tumor thickness of the primary cutaneous melanomas was seen in patients with 25(OH)D s.c. <10 ng/ml (median: 2.55 mm) as compared to >20 ng/ml (median: 1.5 mm), although this difference was not statistically significant (*p* = 0.078). The patients with low 25(OH)D s.c. (<10 ng/ml) had earlier distant metastatic disease (median: 24.37 months) as compared to those with 25(OH)D s.c. >20 ng/ml (median: 29.47 months), although this difference was also not statistically significant (*p* = 0.641) (7). Newton-Bishop et al. reported in the same year a significant association between higher vitamin D level and lower Breslow primary tumor thickness as well as lower rates of relapse and death in a two-stage study (8). In 2014, we published an affiliating investigation (9), demonstrating significantly (*p * =  0.004) lower 25(OH)D s.c. in melanoma patients (median  =  13.6 ng/ml) as compared to controls (median   = 15.6 ng/ml). Primary tumors of patients with low 25(OH)D s.c. (<10 ng/ml) had significantly (*p*  =  0.006) greater Breslow thickness (median: 1.9 mm) as compared to patients with higher levels (>20 ng/ml; median: 1.00 mm). In that study, patients with 25(OH)D s.c. in the lowest quartile had inferior overall survival (OS; median: 80 months) compared with the highest quartile (median: 195 months; *p*  =  0.049).

The experimental support for the concept that vitamin D status is of relevance for progression of melanoma and other malignancies was the demonstration of the almost ubiquitous expression (including in melanocytes, melanoma cells, and many immune cells) of both the vitamin D receptor (VDR) and the activating enzyme (vitamin D-1α-hydroxylase, CYP27B1), which converts serum 25-hydroxyvitamin D [25(OH)D] into the biologically active vitamin D metabolite 1,25-dihydroxyvitamin D [1,25(OH)_2_D] (10, 11).

Vitamin D compounds exert many potent antitumor effects on melanoma and other malignancies that involve regulation of proliferation, differentiation, apoptosis, angiogenesis, stemness of cancer stem cells, tumor microenvironment, and various immune functions (12) *via* a complex network of cellular signaling pathways. Binding and transcriptional activation of VDR by 1,25(OH)_2_D regulate multiple pathways [including VDR-, peroxisome proliferator-activated receptor (PPAR)-, p53-, Wnt/β- (The term Wnt results from the fusion of the name of the *Drosophila* segment polarity gene termed *wingless* and the name of the vertebrate homolog, *integrated or int-1*), catenin-, and mitogen-activated protein kinase (MAPK)- mediated signaling] and functions that are implicated in carcinogenesis, cancer progression, metastatic potential, and immunosurveillance (12). Vitamin D-mediated immunomodulating activities 10,12] that have been widely explored in autoimmune disorders include inhibition of T-helper lymphocytes [often involved in the pathogenesis of immune-related adverse events (iAEs) that complicate ICI treatment], upregulation of Programmed death-ligand-1 (PDL-1) expression on both epithelial and immune cells (suggesting a synergic therapeutic effect in combination with ICIs, for which further investigation is needed), and stimulation of antimicrobial peptides in macrophages and other immune cells (increasing the efficacy of antibody-based cancer therapy) (9, 13).

In summary, an increasing body of evidence now convincingly indicates that 25(OH)D s.c. is of high importance for the clinical outcome in melanoma patients. However, there are limited data of the impact of 25(OH)D s.c. on clinical outcome in advanced melanoma. Considering the growing interest in comparative effectiveness research (CER) and to gain further insights into the relevance of serum 25(OH)D concentrations on prognosis of melanoma, we have now carried out this side-by-side analysis in a subgroup of patients that is at present of particular importance, namely, patients treated for metastasized disease with BRAFi/MEKi (monotherapy with BRAFi—dabrafenib, vemurafenib, encorafenib—and/or combination therapy with BRAFi and MEKi—dabrafenib/trametinib, vemurafenib/cobimetinib, and encorafenib/binimetinib) and/or immunotherapy (mono- and or combination therapy with CTLA-4i—ipilimumab—and/or PD-1i—pembrolizumab, nivolumab).

## Materials and Methods

### Study Design

**Figure 1:**
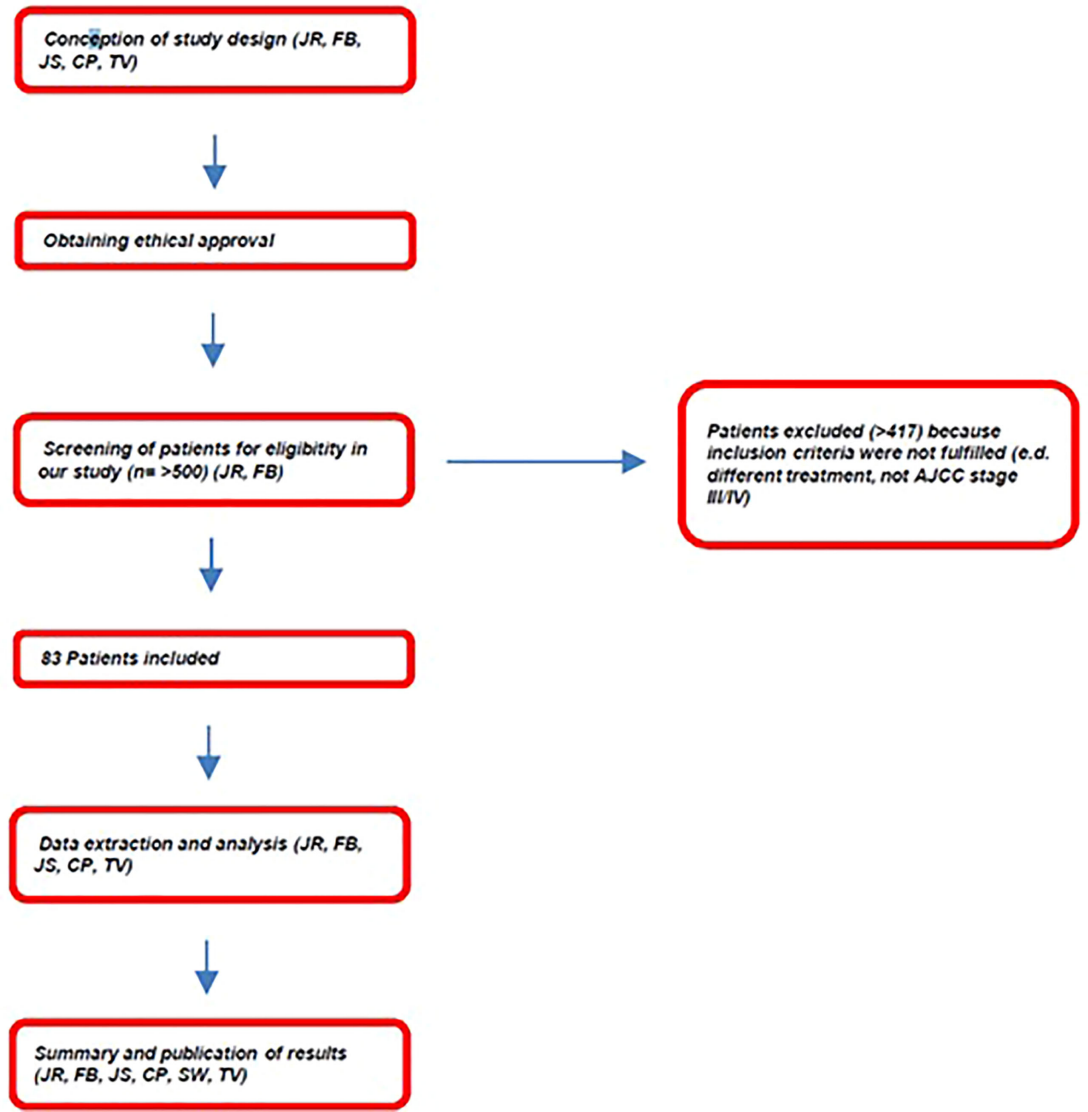
Flowchart of the study design.

This prospective, comparative, non-interventional side-by-side study represents an interim analysis of an ongoing study at the Dermatology Clinic of the Saarland University Medical Center that investigates patients treated for metastasized cutaneous melanoma with either ICIs (CTLA-4i, PD-1i) or BRAFi/MEKi that was performed in agreement with good clinical practice (GCP) criteria, including European Medicines Agency (EMA) Guideline on Good Pharmacovigilance Practices (GVP) Module VIII (14), and that was approved by the Ethical committee of the “Ärztekammer des Saarlandes” (No. 129/18). A flowchart of the study design is shown in **Figure 1**. After screening a cohort of more than 500 patients treated at the Dermatology Clinic of the Saarland University Medical Center for cutaneous melanoma, 83 patients could be included in our study (**Figure 1**).

Inclusion criteria were treatment with CTLA-4i, PD-1i, or BRAFi/MEKi for histologically and clinically confirmed advanced melanoma [stages III and IV according to the 2009 edition of the American Joint Committee on Cancer Cancer (AJCC) Staging Manual] and having given informed consent. Exclusion criteria were defined accordingly. Patient characteristics are shown in **Tables 1** **–** **3**.

**Table 1:** Demographic and clinical data of melanoma patients^1^ included in this study.

	(n=)	OP (weeks)	Age (mean)	Gender	25(OH)D s.c. (ng/ml)^2^		Survival^3^ (weeks)		Tumor load^4^		Adverse events (side effects ratio)^5^	Mutation status (wt/mut)^6^		
					Baseline^2^ (mean)	Average^2^	OS	PFS	LDH (U/L)(Baseline^2^/Average^2^)	S100 (µg/L)		BRAF	NRAS	c-KIT
All patients^1^	83	54.32	63.43	45m 38f	19.61	21.05	100.66/97.00	53.94/34.00	321.6/344.0	0.74/1.02	0.5438	24	22	3
25(OH)D s.c. <10 ng/ml	13	25.30	63.15	7m 6f	7.2	7.36	29.59/19.00	20.30/12.00	419.5/490.1	1.51/2.35	0.6982	3	6	0
25(OH)D s.c. ≥10 ng/ml	70	59.78	63.49	38m 32f	22.05	23.71	112.04/117.00	60.83/48.00	305.0/319.3	0.6/0.77	0.518	21	16	3
BRAF wt	59	53.28	66.53	32m 27f	15.83	20.48	109.29/97.00	54.07/33.00	299.1/330.5	0.7/1.02	0.6156			
25(OH)D s.c. <10 ng/ml	10	24.20	68.10	6m 4f	7.45	7.59	29.92/19.00	14.09/11.00	413.6/491.3	1.60/2.62	0.7680			
25(OH)D s.c. ≥10 ng/ml	49	59.22	66.2	26m 23f	17.58	23.11	121.23/142.00	61.52/48.00	275.2/297.7	0.50/0.69	0.5845			
BRAF mut	24	56.96	55.83	13m 11f	30.02	22.64	83.98/63.00	57.00/60.00	394.3/391.0	0.89/1.02	0.308			
25(OH)D s.c. <10 ng/ml	3	29.00	46.67	1m 2f	6.37	6.58	29.00/16.00	37.00/13.00	N.A.^7^	1.02/1.02	N.A.^7^			
25(OH)D s.c. ≥10 ng/ml	21	61.15	57.14	12m 9f	33.97	25.32	93.40/68.00	60.85/34.00	389.4/385.6	0.87/1.02	0.3265			
Treatment modalities^1^														
BRAF-/MEK- inhibitors^1^	19	62.42	56.05	9m 10f	31.5	22.9	81.77/62.00	54.70/34.00	310.57/287.9	0.24/0.58	0.4062	18	0	0
Immunotherapy^1^														
CTLA-4 and PD-1 inhibitors	16	46.25	48.13	7m 9f	32.68	22.54	82.53/97.00	43.62/48.00	393.5/419.6	1.18/1.34	0.8150	6	4	2
CTLA-4-inhibitors	13	103.23	60.77	4m 9f	12.31	21.66	108.87/78.00	47.97/22.00	270.9/297.7	0.54/0.76	0.6738	1	5	0
PD-1 inhibitors	63	60.56	67.17	34m 29f	16.52	21.44	106.62/97.00	55.44/33.00	294.3/318.3	0.59/0.91	0.4844	12	19	1
	**Patients with Metastases before therapy (n=)**					**other**	**Total number of metastasized organs**	**Total number of metastasized organs/number of patients**						
	**(n=)**	**brain**	**lung**	**liver**	**skin/soft tissue**									
All patients^1^	82	19	50	25	29	39	162	1.95						
25(OH)D s.c. < 10 ng/mL	13	5	10	5	3	6	29	2.23						
25(OH)D s.c. ≥ 10 ng/mL	69	14	40	20	26	33	133	1.92						
BRAF and MEK inhibitors	19	5	8	4	3	13	33	1.7						
Immunotherapy^1^														
CTLA-4 and PD-1 inhibitors	16	5	7	5	2	11	30	1.88						
CTLA-4-inhibitors	13	3	9	3	6	5	26	2						
PD-1 inhibitors	63	16	40	16	27	30	129	2.04						

^1^For treatment modalities, please see **Table 2**.

^2^25(OH)D serum concentrations were investigated as described in the Methods. In short, 25(OH)D serum concentration was either assessed when starting therapy with ICIs or BRAFi/MEKi [“baseline 25(OH)D”] or calculated as average value of all 25(OH)D serum concentrations that were measured for each patient during the complete observation period [“average 25(OH)D”].

^3^Mean and Median Survival Estimates was assessed as described in the Methods.

^4^Tumor load was estimated by analyzing serum concentrations of LDH and S100 protein, as described in the Methods.

^5^Adverse events were assessed according to CTCAE criteria as described in the Methods.

^6^Mutation status was assessed as described in the Methods.

^7^Missing data due to small case number.

BRAF, B-rapidly accelerated fibrosarcoma; CTCAE, common terminology criteria for adverse events; CTLA, cytotoxic T-lymphocyte-associated protein; LDH, lactate dehydrogenase; MEK, mitogen-activated protein kinase kinase; Mut, mutated; OP, observation period; 25(OH)D s.c., 25(OH)D serum concentration; OS, overall survival; PD, programmed cell death protein; PFS, progress-free survival; TL, tumor load; wt, wild-type.

Rounding errors may occur in the data table. The number of patients may differ from the total sample size owing to missing data.

**Table 2:** Treatment modalities of melanoma patients included in this study.

Treatment	(n=)	OS (weeks) (mean/median)	Age (years) (mean/median)	Gender	Treatment modalities		
					Dosage	Frequency	Duration
All patients	83	100.66/97.00	63.43/65.00	45m 38f			
BRAF-/MEK- inhibitors							
BRAF and MEK inhibitors	19	81.77/62.00	56.00/55.00	9m 10f			
Dabrafenib (Tafinlar©)/Trametinib (Mekinist©)	16	63.69/62.00	54.93/53.00	9m 7f	150 mg/2 mg	2x daily/1x daily	until disease progression or unacceptable toxicity
Vemurafenib (Zelboraf©)/Cobimetinib (Cotellic©)	3!	128.33/120.00	62.00/56.00	0m 3f	960 mg/60 mg	2× daily/1× daily	until disease progression or unacceptable toxicity
Immunotherapy							
CTLA-4 and PD-1 inhibitors							
Ipilimumab (Yervoy ©)/Nivolumab (Optivo©)	16	82.53/97.00	48.13/55.0	7m 9f	3 mg/kg ipilimumab	Ipilimumab 4x (every 3 weeks),	until disease progression or unacceptable toxicity
					1 mg/kg nivolumab	then nivolumab (every 2 weeks)	
CTLA-4-inhibitor							
Ipilimumab (Yervoy ©) monotherapy	13	108.88/78.00	60.77/60.0	4m 9f	3 mg/kg	every 3 weeks 4x	
PD-1 inhibitors	63	106.62/97.00	67.17/69.0	34m 29f			
Pembrolizumab (Keytruda ©) monotherapy	47	110.20/97.00	67.64/70.0	26m 21f	2 mg/kg	every 3 weeks	until disease progression or unacceptable toxicity
Nivolumab (Optivo©) monotherapy	19	88.32/67.00	64.36/68.0	9m 10f	3 mg/kg	every 2 weeks	until disease progression or unacceptable toxicity

BRAF, B-rapidly accelerated fibrosarcoma; CTLA, cytotoxic T-lymphocyte-associated protein; MEK, mitogen-activated protein kinase; OP, observation period; PD, programmed cell death protein.

Rounding errors may occur in the data table. The number of patients may differ from the total sample size owing to missing data.

**Table 3:** Summary of key study results.

	Overall survival (Kaplan–Meier analysis)						Risk to die (Cox regression)									
								
*Baseline 25(OH)D s.c.*																
All patients <10 ng/ml vs. ≥10 ng/ml	65.79 vs. 116.07	[41.33–90.26] vs. [86.33–145.81]	0.008		25 vs. 57		0.413			0.21 – 0.82			0,01			82
BRAFmut <10 ng/ml vs. ≥10 ng/ml	51.00 vs. 105.86	[18.41–83.59] vs. [48.28–163.44]	0.173		6 vs. 17		0.473			0.16 – 1.43			0.185			23
BRAFwt <10 ng/ml vs. ≥10 ng/ml	81.14 vs. 118.45	[47.66–114.61] vs. [82.45–154.45]	0.046		19 vs. 40		0.421			0.18 – 1.01			0.053			59
*Average 25(OH)D s.c.*																
All patients <10 ng/ml vs. ≥10 ng/ml^1^	29.59 vs. 112.04	[15.98–43.21] vs. [87.18–136.91]	0.000001		13 vs.69		0.179			0.08 – 0. 39			0.000016			82
BRAFmut <10 ng/ml vs. ≥10 ng/ml	29.00 vs. 93.40	[0.00–62.63] vs. [52.02–134.79]	0.045		3 vs. 20		0.275			0.07 – 1.07			0.063			23
BRAFwt <10 ng/ml vs. ≥10 ng/ml	29.92 vs. 121.53	[13.85–46.00] vs. [90.94–152.13]	0.000012		10 vs. 49		0.148			0.06 – 0.4			0.000134			59
	**Risk for progress (Cox regression)**															
	**HR**	**95% CI**	**p-value**		**(n=)**											
*Baseline 25(OH)D s.c.*																
All patients <10 ng/ml vs. ≥10 ng/ml	0.429	0.23–0.82	0.01		82											
*Average 25(OH)D s.c.*																
All patients <10 ng/ml vs. ≥10 ng/ml	0.237	0.11–0.50	0.000159		82											
BRAFmut <10 ng/ml vs. ≥10 ng/ml^1^	0.565	0.12–2.74	0.479		21											
BRAFwt <10 ng/ml vs. ≥10 ng/ml	0.161	0.07–0.39	0.000061		59											
		**average LDH s.c.**	**95% CI**		***p*-value**	**(n=)**			**average S100P s.c.**	**95% CI**	***p*-value**					**(n=)**
		**(U/l)**							**(µg/l)**							
*Average 25(OH)D s.c.*																
All patients <10 ng/ml vs. ≥10 ng/ml		-170.83	-312.15 - -29.51		0.019	75			-1.58	-3.04 to -0.12	0.034					74
BRAFmut <10 ng/ml vs ≥10 ng/ml		-92.45	-878.52 - 693.63		0.805	16			0.005	-4.73 – 4.74	0.998					15
BRAFwt <10 ng/ml vs. ≥10 ng/ml		-193.67	-305.96 - -81.39		0.001	58			-1.922	-3.44 to -0.409	0.014					58
**Adverse Events Ratio**	**AEs regression coefficient**	**95% CI**	***p*-value**		**(n=)**											
*Baseline 25(OH)D s.c.*																
All patients <10 ng/ml vs. ≥10 ng/ml	-0.10	-0.37 - 0.16	0,449		76											
*Average 25(OH)D s.c.*																
All patients <10 ng/ml vs. ≥10 ng/ml	-0.18	-0.53 - 0.17	0.303		76											
BRAFmut <10 ng/ml vs. ≥10 ng/ml	0.326	-0.304 - 0.9574	0.289		17											
BRAFwt <10 ng/ml vs. ≥10 ng/ml	-0.184	-0.58 - 0.213	0.358		58											
	Per increase of 1 ng/ml 25(OH)D s.c. (baseline)					Per increase of 1 ng/ml 25(OH)D s.c. (average										
	HR	95%CI	p-value		(n=)	HR	95%CI		p-value	(n=)						
All patients	0.99	0.96 - 1.02	0.657		79	0.96	0.92 - 1.00		0.079	80						
BRAFwt	n.s.	n.s.	n.s.		n.s.	0.96	0.91 - 1.02		0.169	59						
BRAFmut	n.s.	n.s.	n.s.		n.s.	0.97	0.91 - 1.04		0.851	21						
**Linear regression**																
	Per increase of 1 ng/ml 25(OH)D s.c. (baseline)								Per increase of 1 ng/ml (average) 25(OH)D s.c.							
	baseline LDH				baseline S100P				average LDH				average S100P			
	(U/l)	95%CI	p-value	(n=)	(µg/l)	95%CI	p-value	(n=)	(U/l)	95%CI	p-value	(n=)	(µg/)	95%CI	p-value	(n=)
All patients	-0.71	-2.01 - 0.59	0.278	74	-0.004	-0.015 to 0.007	0.449	72	-3.84	-7.34 to -0.33	0.033	75	-0.032	-0.07 to 0.01	0.106	73
BRAFwt	-3.62	-8.37 - 1.13	0.133	56	-0.038	-0.09 to -0.01	0.114	55	-5.98	-10.78 to -1.18	0.016	58	-0.058	-0.12 to 0.01	0.075	58
BRAFmut	-0.834	-3.02 - 1.36	0.431	17	-0.003	-0.02 to 0.01	0.668	16	-3.21	-10.53 to 4.11	0.365	16	-0.02	-0.09 to 0.05	0.527	14
**Linear regression**																
Per increase of 1 ng/ml 25(OH)D s.c. (average)																
Adverse events (AEs)	AE ratio	95%CI	p-value	(n=)												
All patients	-0.005	-0.01 - 0.004	0.295	75												
BRAFwt	-0.012	-0.03 - 0.004	0.137	58												
BRAFmut	0.001	-0.005 – 0.007	0.765	16												
**Risk to die (Cox regression)**	**HR**	**95% CI**	**p-value**													
Average 25(OH)D s.c. <10 ng/ml vs. ≥10 ng/ml, adjusted for																
BRAFmut vs. N-RASmut vs. BRAFwt + NRASwt	0.138	0.059–0.352	0.000004													
Average LDH s.c.	0.306	0.108–0.868	0.026													
Average S100P s.c.	0.225	0.085 - 0.599	0.003													
BRAFmut/N-RASmut/average LDH s.c./average S100P s.c.	0.177	0.058–0.544	0.003													

AE, adverse event; BRAFmut, BRAFmutant; BRAFwt, BRAF wild type; Ci, confidence interval; HR, hazard ratio; LDH, lactate dehydrogenase; n.s., not stated; OP, observation period; OR, odds ratio; OS, overall survival; PFS, progression-free survival; s.c., serum concentration.

Rounding errors may occur in data table. Number of patients may differ from total sample sizes due to missing data. ^1^Data need to be interpreted with caution because the proportional hazard assumption is not fulfilled. ^2^Regression coefficient.

Data obtained during the observation period (OP) [January 2014 until death or August 2018 (the end of the OP), whichever came first] were collected from patient records and maintained in a specifically designed spreadsheet. Patient records/information was pseudonymized prior to analysis. According to their vitamin D status [“baseline 25(OH)D”: 25(OH)D s.c. measured immediately before starting treatment with either ICIs (CTLA-4i, PD-1i) or BRAFi/MEKi; “average 25(OH)D”: mean values of all 25(OH)D s.c. that were measured for each patient during the OP], patients were divided into different subgroups. Primary outcome measures were OS [time from study entry (start of treatment with CTLA-4i, PD-1i, or BRAFi/MEKi) to date of death or censored as date of last follow-up if still alive at conclusion of follow-up], progress-free survival [PFS; time from study entry (start of treatment with CTLA-4i, PD-1i, or BRAFi/MEKi) to date of progress of melanoma, or censored as date of last follow-up if without progress], tumor load (TL), and frequency of adverse events (AEs).

### Extraction and Analysis of Clinical and Laboratory Data

A total of 83 patients were included (**Table 1**), with clinical and laboratory data being obtained from each follow-up. All blood samples were analyzed at the Department of Clinical Chemistry and Laboratory Medicine of the Saarland University Medical Center in Homburg. Vitamin D levels were tested as 25(OH)D s.c. (ng/ml; 1 ng/ml  =  2.5 nmol/L) using LIAISON^®^ 25-OH Vitamin D Total (DiaSorin, Dietzenbach, Germany). Here, 25(OH)D s.c. was measured at the beginning of the OP [“baseline 25(OH)D”] and at each follow-up to calculate for every patient an individual average 25(OH)D s.c. [“average 25(OH)D”] during the OP. The lower detection limit of this assay is at 4 ng/ml, and the within-pair coefficient of variation (CV) of this assay was published 4.9% using blinded quality control samples (15).

Concentrations of lactate dehydrogenase (LDH) and S100 protein (S100P) were tested in blood samples using standard assays every month as an estimate of the TL. Disease stage was determined according to the 2009 edition of the American Joint Committee on Cancer Cancer (AJCC) Staging Manual.

Clinical data extracted included results of routinely performed staging examinations [in general every 3-month computer tomography (CT) or magnetic resonance imaging (MRI) of head, thorax, abdomen] that were classified in four categories considering the Response Evaluation Criteria in Solid Tumors (RECIST) criteria (16) as follows: complete remission (CR), partial remission (PR), stable disease (SD), and progressive disease (PD). OP started with the beginning of therapy and ended either by death or by end of the study in August 2018, whichever came first. Additionally, clinical data extracted included frequency and severity of AEs that were assessed according to GCP criteria, considering the Common Terminology Criteria for Adverse Events in different categories as published previously (CTCAE v5.0; https://ctep.cancer.gov/protocolDevelopment/electronic_applications/ctc.htm#ctc_50).

### Statistical Analysis

Classification of vitamin D deficiency [often defined as 25(OH)D s.c. <20 ng/ml (50 nmol/L)] remains controversial and often depends on the type of laboratory testing used. In line with our previous studies (7, 10), we defined by convention vitamin D deficiency as 25(OH)D s.c. <20–≥10 ng/ml and severe deficiency as <10 ng/ml. For statistical analysis, we dichotomized vitamin D at 10 ng/ml.

For assessing OS, Kaplan–Meier analysis with log-rank comparison testing and Cox regression were performed in subgroups characterized by baseline or average 25(OH)D s.c. <10 ng/ml as compared to ≥10 ng/ml. To detect potentially disturbing variables, Cox regression was adjusted with potentially interfering factors, including metastases of liver or kidneys (before and during therapy), representing important organs of the vitamin D endocrine system, sex, age, and mutation status of melanomas (*b-raf*, *c-kit*, *n-ras*). Metastases were identified by full-body CT or MRI examinations that were performed before and every 3 months during therapy to evaluate treatment outcome.

Adjustment with other potentially interfering factors, including history of other malignancies, diabetes mellitus, hyperlipidemia, hypercholesterinemia, stroke, coronary heart disease, heart insufficiency, heart rhythm disorders, hypertension, kidney, thyroid or liver disease, asthma bronchiale, and ethanol and nicotine abuses, was performed using Cox regression modeling.

To analyze the impact of 25(OH)D s.c. on melanoma progress, Cox regression was performed with “time to progress” as dependent variable and 25(OH)D s.c. (<10 ng/ml as compared to ≥10 ng/ml) before [baseline 25(OH)D s.c.] and during [average 25(OH)D s.c.] therapy as predictor. Status was either progress or no progress.

In order to analyze whether 25(OH)D s.c. is associated with distinct types of melanoma progression, logistic regression was performed using the presence of brain, lung, or liver metastases before therapy as dependent variable and baseline 25(OH)D s.c. as predictor.

For assessing the association of 25(OH)D s.c. with TL, s.c. of LDH and S100P was determined once a month. Linear regression analysis (ANOVA) was performed with baseline (at initiation of treatment) and average LDH and S100P serum levels during therapy as dependent variables and baseline and average 25(OH)D s.c. during therapy as a predictor.

To investigate the association of 25(OH)D s.c. with the frequency and severity of well-defined AEs during the OP, a score (ratio of frequency of AEs/time) was calculated and used as dependent variable in linear regression analysis (ANOVA) with baseline and average 25(OH)D s.c. as predictors. Heretofore, the total number of any AE during the OP that was assessed according to GCP criteria was recorded and summarized for every individuals. Because the length of the OP differed between individual patients, for each patient, the total number of AEs was divided through the duration of the OP (days), resulting in an AE ratio that was used as a score to assess the frequency of AEs.

All analyses (except for recursive partitioning) were performed using SPSS (IBM SPSS Statistics 24; RRID : SCR_019096). Kaplan–Meier survival analysis was performed, Cox regression models were built to estimate hazard ratios (HRs), and adjusted survival curves were plotted. All *p*-values were two sided. *P*-values less than 0.05 were considered statistically significant.

## Results

### Low Serum 25(OH)D Concentrations Predict Poor Survival in Melanoma Patients Treated With Immune Checkpoint Inhibitors and/or BRAFi/MEKi

First, we analyzed in this cohort of melanoma patients as a primary outcome the association of OS with vitamin D status [assessed as either the 25(OH)D s.c. at the time when starting therapy with ICIs or BRAFi/MEKi (“baseline 25(OH)D”) or as the average value of all 25(OH)D s.c. that was measured for each patient during the complete OP (“average 25(OH)D”)].

**Figure 2:**
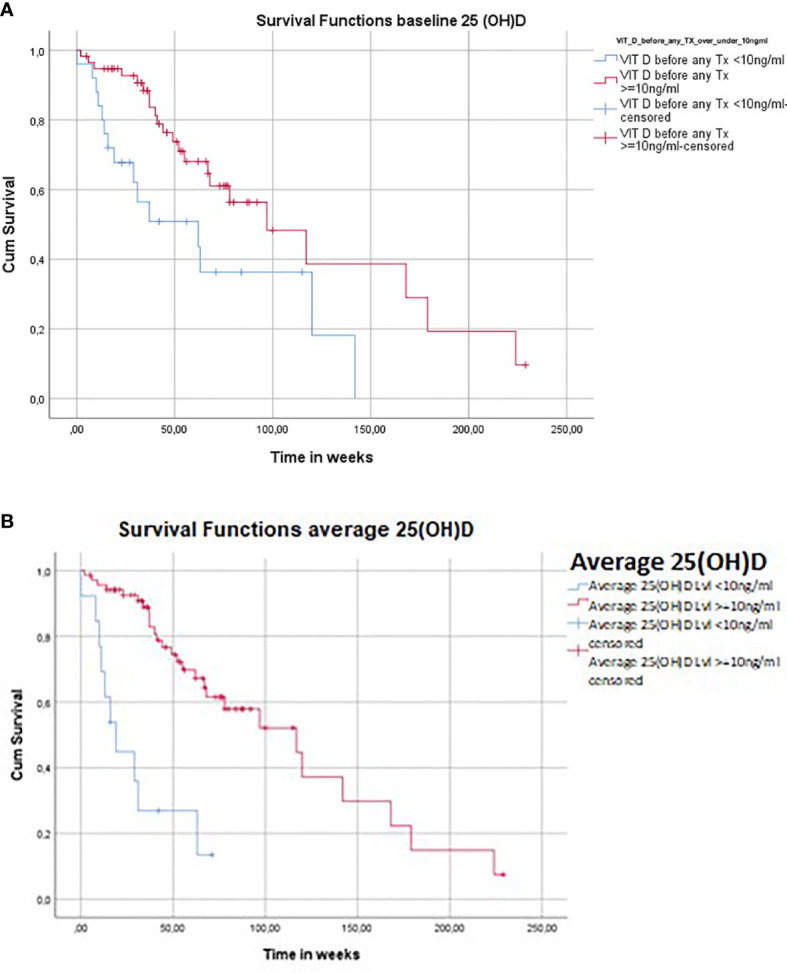
Low 25(OH)D serum concentrations (s.c.) predict poor overall survival (OS) in melanoma patients treated for advanced disease with immunotherapy and/or BRAF/MEK inhibitors. **(A)** Association of OS with baseline 25(OH)D s.c. in patients treated for advanced melanoma with ICIs and/or BRAF/MEK inhibitors. Cohort divided in subgroups according to 25(OH)D s.c. measured immediately before starting therapy with ICIs and/or BRAF/MEK inhibitors [“baseline 25(OH)D”]. Kaplan–Meier analysis shows a significant difference in survival, with only 50.8% of individuals in the subgroup of severely vitamin D-deficient patients [defined as 25(OH)D s.c. <10 ng/ml] being alive after 1 year of the observation period (OP) as compared to 71.0% in the subgroup of patients with 25(OH)D s.c. ≥10 ng/ml (*p* = 0.008). The mean OS (100.66 weeks in all patients included in this study) was almost 2-fold increased in the subgroup of patients with baseline 25(OH)D s.c. ≥10 ng/ml (116 weeks) as compared to the subgroup of patients with baseline 25(OH)D s.c. <10 ng/ml (66 weeks) (*p* = 0.008). Rounding error may occur. Number of patients may differ from total sample sizes due to missing data. **(B)** Association of OS with average 25(OH)D s.c. in patients treated for advanced melanoma with ICIs and/or BRAF/MEK inhibitors. Cohort of melanoma patients divided in subgroups according to average values of all 25(OH)D serum concentrations that were measured for each patient during the observation period [“average 25(OH)D”]. Kaplan–Meier analysis shows a significant difference in OS, with only 26.9% of individuals in the subgroup of vitamin D-deficient patients [average 25(OH)D s.c. <10 ng/ml] being alive after 1 year of the OP as compared to 77.2% in the subgroup of patients with average 25(OH)D serum concentrations ≥10 ng/ml (*p* = 0.000001). After 2 years, 0% and 52.1%, and after 4 years, 0% and 14.9% of individuals were alive in the subgroups of patients with average 25(OH)D s.c. <10 ng/ml and ≥10 ng/ml, respectively (*p* = 0.000001). The mean OS (100 weeks in all patients included in this study) was almost 4-fold increased in the subgroup of patients with average 25(OH)D s.c. ≥10 ng/ml (112 weeks) as compared to the subgroup of patients with average 25(OH)D s.c. <10 ng/ml (29 weeks) (*p* = 0.000001). Rounding error may occur. Number of patients may differ from total sample sizes due to missing data. BRAF, B-rapidly accelerated fibrosarcoma; ICI, immune checkpoint inhibitor; LDH, lactate dehydrogenase; MEK, mitogen activated protein kinase; OS, overall survival.

OS (100.66 weeks in all patients included in this study) was almost 2-fold (116.07 vs. 65.79 weeks; median 97.00 vs. 62,00 weeks, *p* = 0.008; **Figure 2A** and **Tables 1**, **3**) and 4-fold (112 vs. 30 weeks, *p* = 0.000001; **Figure 2B** and **Tables 1**, **3**) higher in patients with baseline and average 25(OH)D s.c. ≥10 ng/ml as compared with <10 ng/ml, respectively.

Cox regression showed that during the complete OP (228 weeks/mean OP 54.31 weeks), the risk to die was in patients with baseline and average 25(OH)D s.c. ≥10 ng/ml reduced by 59% [hazard ratio (HR) 0.413, *p* = 0.011] and 82% (HR 0.179, *p* = 0.000016), respectively, as compared to patients with 25(OH)D s.c. <10 ng/ml (**Table 3**).

Analyzing patients being alive after 1 year of the OP, there was a significant difference in OS (Kaplan–Meier analysis; **Figures 2A, B**) comparing individuals with 25(OH)D s.c. <10 ng/ml vs. ≥10 ng/ml [50.8% vs. 71.0% for “baseline 25(OH)D”; *p* = 0.008; and 26.9% vs. 77.2% for “average 25(OH)D”; *p* = 0.000001, respectively]. Comparing patients with average 25(OH)D s.c. <10 ng/ml vs. ≥10 ng/ml, 0% vs. 52.1% and 0% vs. 14.9% of individuals were alive after 2 and 4 years, respectively (**Figure 2A**; *p* = 0.000001).

In line with these findings, the risk to die was reduced in patients with average 25(OH)D s.c. ≥10 ng/ml (mean 23.71 ng/ml) as compared with vitamin D-deficient patients after adjusting for various parameters, including *BRAF*/*N-RAS* mut (HR: 0.138, *p* = 0.000004) and markers of TL such as average LDH (HR 0.306, *p* = 0.026) and S100P (HR 0.225, *p* = 0.003; **Table 3**). If all these parameters were taken into account for adjusting Cox regression model, there was still a significant reduction in HR (0.177, *p* = 0.003) for patients with average 25(OH)D s.c. ≥10 ng/ml compared to those with 25(OH)D <10 ng/ml.

We then looked at the association of 25(OH)D s.c. with OS in subgroups of patients with *BRAF* wild-type (wt) and *BRAF* mutant (mut) melanomas. However, these results need to be interpreted with caution because the study size is too small to guarantee reliability. Figures demonstrating the findings of these subgroup analyses are therefore not presented in the main text but are included as supplementary information. In summary, in patients with *BRAF*mut melanomas, OS was reduced in patients with baseline 25(OH)D s.c. <10 ng/ml (mean OS 51.0 weeks, median OS 37.00) as compared to baseline 25(OH)D s.c. ≥10 ng/ml (mean OS 105.86 weeks, median OS 117.00 weeks), respectively (**Figure S1**; *p* = 0.173). After 1 year, 50.0% and 71.3%, after 2 years, 16.7% and 51.0%, after 3 years, 0% and 25%, and after 4 years, 0% and 25% of patients with *BRAF*mut melanomas and with baseline 25(OH)D s.c. <10 ng/ml, as compared to ≥10 ng/ml, respectively (**Figure S1**; *p* = 0.173), were alive.

In patients with *BRAF*mut melanomas, OS was also reduced in patients with average 25(OH)D s.c. <10 ng/ml (mean OS 29.0 weeks, median OS 16.00) as compared to patients with average 25(OH)D s.c. ≥10 ng/ml (mean OS 93.4 weeks, median OS 68.00 weeks), respectively (**Figure S2**; *p* = 0.045). After 1 year, 33.3% and 69.6%, after 2 years, 0% and 46.4%, after 3 years, 0% and 15.5%, and after 4 years, 0% and 15.5% of individuals were alive in the subgroups of patients with average 25(OH)D s.c. <10 ng/ml and ≥10 ng/ml, respectively (**Figure S2**; *p* = 0.045). For patients with *BRAF*mut melanomas, risk to die was during the complete OP reduced by 52.7% in individuals with baseline 25(OH)D s.c. ≥10 ng/ml as compared with severely vitamin D-deficient patients [baseline 25(OH)D s.c. <10 ng/ml] (**Table 3** and **Figure S3**; *p* = 0.185).

For patients with *BRAF*mut melanomas, risk to die was during the complete OP reduced by 72.5% in individuals with average 25(OH)D s.c. ≥10 ng/ml as compared with severely vitamin D-deficient patients (**Figure S4**; *p* = 0.063).

In patients with *BRAF*wt melanomas, after 1 year, 51.0% and 70.9%, after 2 years, 51.0% and 43.9%, after 3 years, 0% and 43.9%, and after 4 years, 0% and 14.6% of individuals with baseline 25(OH)D s.c. <10 ng/ml, as compared to ≥10 ng/ml, respectively, were alive (**Figure S5**; *p* = 0.046).

Comparing BRAFwt melanoma patients with 25(OH)D s.c. <10 ng/ml vs. ≥10 ng/ml, after 1 year, 24.0% vs. 73.1%, after 2 years, 0% vs. 52.5%, after 3 years, 0% vs. 39.4%, and after 4 years, 0% vs. 13.1% of individuals were alive, respectively (**Figure S6**; p = 0.000012).

For patients with *BRAF*wt melanomas, risk to die was during the complete OP reduced by 57.9% in individuals with baseline 25(OH)D s.c. ≥10 ng/ml as compared with severely vitamin D-deficient patients (**Table 3** and **Figure S7**; *p* = 0.053).

In the subgroup of patients with *BRAF*wt melanomas, risk to die during the complete OP (228 weeks/mean 54.31 weeks) was reduced by 85.2% in individuals with average 25(OH)D s.c. ≥10 ng/ml as compared with severely vitamin D-deficient patients (**Figure S8** and **Table 3**, *p* = 0,000134).

When potentially interfering factors, including location of metastases (e.g., in liver or kidneys, representing important organs for vitamin D metabolism), sex, age, history of other malignancies, diabetes mellitus, hyperlipidemia, hypercholesterinemia, stroke, coronary heart disease, heart insufficiency, heart rhythm disorders, hypertension, kidney, thyroid or liver disease, asthma bronchiale, ethanol and nicotine abuse, were assessed to adjust Cox regression modeling, mutations in *BRAF*, n*-ras*, and c-*kit* genes were identified, and Cox regression model was adjusted using these variables.

Because of the limitations caused by the relatively small study size, we did not analyze the association of 25(OH)D s.c. with OS in subgroups of patients treated with different therapies. The relatively small numbers of patients in subgroups with different therapies would have resulted in too low power for statistical analysis.

### Low Serum 25(OH)D Concentrations Predict Poor Progress-Free Survival in Melanoma Patients Treated With Immune Checkpoint Inhibitors and/or BRAFi/MEKi

**Figure 3:**
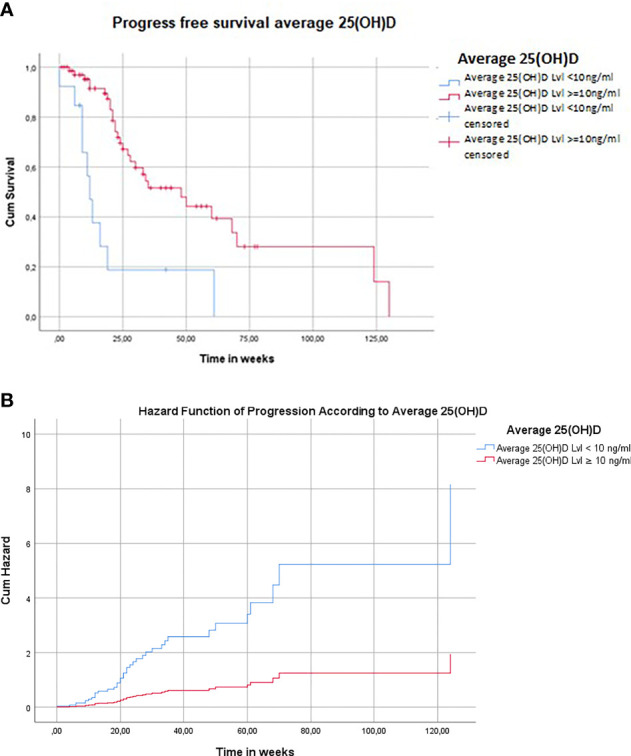
Low 25(OH)D s.c. predict poor progress-free survival (PFS) in melanoma patients treated for advanced disease with ICIs and/or BRAF/MEK inhibitors. **(A)** Association of PFS with average 25(OH)D s.c. in patients treated for advanced melanoma with ICIs and/or BRAF/MEK inhibitors. The mean PFS (53.94 weeks in all patients included in this study) was 3-fold increased in the subgroup of patients with average 25(OH)D s.c. ≥10 ng/ml (60.83 weeks) as compared to the subgroup of patients with average 25(OH)D s.c. <10 ng/ml (20.3 weeks) (*p* = 0.000039). Rounding error may occur. Number of patients may differ from total sample sizes due to missing data. **(B)** Association of hazard function of PFS with average 25(OH)D s.c. in patients treated for advanced melanoma with ICIs and/or BRAF/MEK inhibitors. During the complete observation period (228 weeks; mean 54.31 weeks), the risk for a progress of the disease was by 76.3% reduced in patients with average 25(OH)D s.c. ≥10 ng/ml as compared to severely vitamin D-deficient individuals [mean 25(OH)D s.c. <10 ng/ml] (HR 0.237, *p* = 0,000159). An increase in average 25(OH)D s.c. of 1 ng/ml was associated with a 3.9% reduced risk for a progress of the disease (HR 0.961, *p* = 0.044). Rounding error may occur. Number of patients may differ from total sample sizes due to missing data.

PFS (53.94 weeks in all patients included in this study) was 3-fold increased in patients with average 25(OH)D s.c. ≥10 ng/ml (60.83 weeks) as compared to patients with average 25(OH)D s.c. <10 ng/ml (20.3 weeks) (**Figure 3A**; *p* = 0.000039).

**Figure 4:**
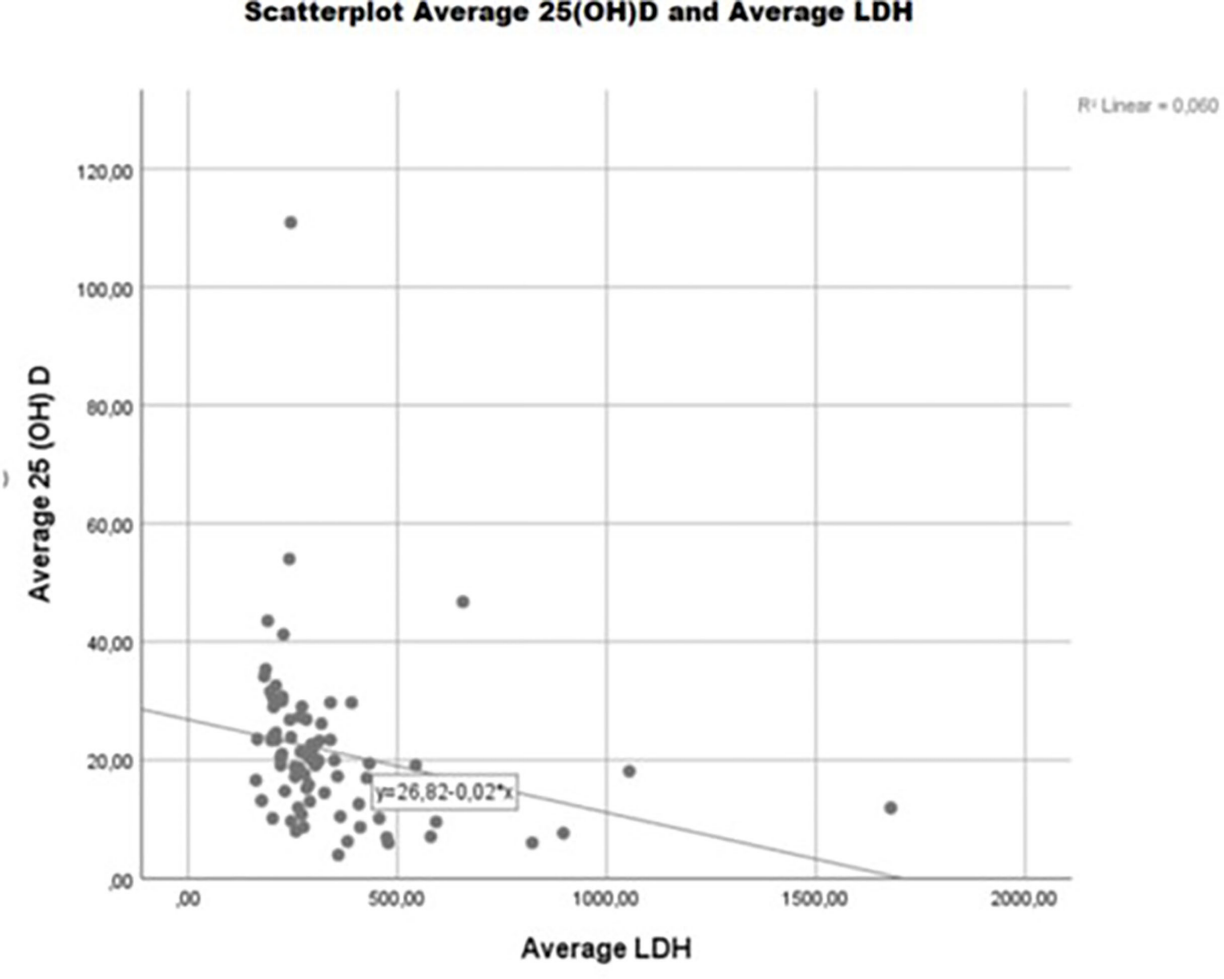
Vitamin D status is associated with LDH s.c. in advanced melanoma patients treated with BRAF/MEK and/or immune checkpoint inhibitors. During the complete OP, LDH s.c. was significantly lower (-170.826 U/l; *p* = 0.019) in patients with average 25(OH)D s.c. ≥10 ng/ml as compared to severely vitamin D-deficient individuals [average 25(OH)D s.c <10 ng/ml]. In general, an increase in average 25(OH)D s.c. of 1 ng/ml was associated with a significant reduction of LDH s.c. of 3.84 U/l (*p* = 0.034). Rounding error may occur. Number of patients may differ from total sample sizes due to missing data.

During the complete OP (228 weeks; mean 54.31 weeks), the risk for disease progression was by 76.3% reduced in patients with average 25(OH)D s.c. ≥10 ng/ml as compared to vitamin D severely deficient individuals [average 25(OH)D s.c. <10 ng/ml] (**Table 3** and **Figure 4**; *p* = 0.000159). An increase in average 25(OH)D s.c. of 1 ng/ml was associated with a reduced risk of 4.0% for a disease progression (**Table 3** and **Figure 3B**; *p* = 0.037).

We then looked at the association of 25(OH)D s.c. with PFS in subgroups of patients with *BRAF*wt and *BRAF*mut melanomas. However, these results need to be interpreted with caution because the study size is too small to guarantee reliability. Figures demonstrating the findings of these subgroup analysis are therefore not presented in the main text but are included as supplementary information. In *BRAF*mut patients with average 25(OH)D s.c. ≥10 ng/ml, the risk for a progress of the disease was not significantly reduced by 43.5% as compared to severely vitamin D-deficient individuals [average 25(OH)D s.c. <10 ng/ml] (**Table 3** and **Figure S9**; *p* = 0,479). There was a trend that in individuals with *BRAF*mut melanomas, an increase of 1 ng/ml baseline 25(OH)D was associated with a 0.5% reduced risk for progressive disease (**Table 3**; HR 0.995, *p* = 0.851).

In patients with *BRAF*wt melanomas and with average 25(OH)D s.c. ≥10 ng/ml, the risk for a progress of the disease during the complete OP was significantly reduced by 83.9% as compared to severely vitamin D-deficient individuals [average 25(OH)D s.c. <10 ng/ml] (**Table 3**; *p* = 0.000061). In patients with *BRAF*wt melanomas, an increase of 1 ng/ml average 25(OH)D was associated with a 6.6% reduced risk for progressive disease during the complete OP (*p* = 0.007).

### In Melanoma Patients Treated With BRAF/MEK and/or Immune Checkpoint Inhibitors, Serum 25(OH)D Concentrations Are Associated With Tumor Load

In melanoma patients, vitamin D status was associated with TL (measured/estimated as LDH and S100P s.c.) before and along with treatment with BRAF/MEK and/or ICIs (**Figure 4**).

Before therapy, S100P s.c. in patients with baseline 25(OH)D s.c. ≥10 ng/ml was not significantly lowered (-0.524 µg/l) as compared to severely vitamin D-deficient individuals (**Table 3**; *p* = 0.251).

However, there was a trend that an increase of 1 ng/ml baseline 25(OH)D was associated with a non-significant decrease of S100P (0.004 µg/l; **Table 3**; *p* = 0.449) and of LDH (0.713 U/l; **Table 3**; *p* = 0.278) s.c.

During the complete OP, there was significantly lower S100P (**Table 3**; -1.583 µg/l; *p* = 0.034) and LDH (**Table 3**; -170.826 U/l; *p* = 0.019) s.c. in patients with average 25(OH)D s.c. ≥10 ng/ml as compared to severely vitamin D-deficient [average 25(OH)D s.c <10 ng/ml] individuals.

In general, an increase in average 25(OH)D s.c. of 1 ng/ml was associated with a significant reduction of LDH s.c. of 3.86 U/l (**Table 3** and **Figure 4**; *p* = 0.034) and a non-significant reduction of S100P s.c. of 0.03 µg/l (**Table 3**; *p* = 0.106).

We then looked at the association of 25(OH)D s.c. with markers for TL in subgroups of patients with *BRAF*wt and *BRAF*mut melanomas. Because of the small study size, these results need to be interpreted with caution. *BRAF*wt patients, with average 25(OH)D s.c. ≥10 ng/ml, had significantly lower (**Table 3**; -1.922 µg/l) S100P s.c. as compared to severely vitamin D-deficient individuals [average 25(OH)D s.c. <10 ng/ml] (**Table 3**; *p* = 0.014).

In comparison, patients with *BRAF* mutation (*BRAF*mut), with average 25(OH)D s.c. ≥10 ng/ml had non-significantly higher S100P s.c. (**Table 3**; +0.005 µg/l, *p* = 0.998) as compared to severely vitamin D-deficient [average 25(OH)D s.c. <10 ng/ml] individuals.

In *BRAF*wt individuals, during the complete OP, LDH s.c. was significantly lower (**Table 3**; -193.673 U/l, *p* = 0.001) in patients with average 25(OH)D s.c. ≥10 ng/ml as compared to severely vitamin D-deficient individuals [average 25(OH)D s.c. <10 ng/ml].

Analyzing individuals with *BRAF*mut melanomas during the complete OP, LDH s.c. was markedly lower (**Table 3**; -92,448 U/l, *p* = 0,805) in patients with average 25(OH)D s.c. ≥10 ng/ml as compared to severely vitamin D-deficient individuals [average 25(OH)D s.c. <10 ng/ml].

In individuals with *BRAF*mut melanomas, an increase in baseline and average 25(OH)D s.c. of 1 ng/ml was associated with a trend for reduced s.c. of LDH {**Table 3**; -0.834 U/l [per increase in baseline 25(OH)D s.c. of 1 ng/ml], *p* = 0.431; and -3.21 U/l [per increase in average 25(OH)D s.c. of 1 ng/ml], *p* = 0.365, respectively} and S100P {-0.003 µg/l [per increase in baseline 25(OH)D s.c. of 1 ng/ml], *p* = 0.668; and -0.02 µg/l [per increase in average 25(OH)D s.c. of 1 ng/ml] during the complete OP, *p* = 0.527, respectively}.

In individuals with *BRAF*wt melanomas, an increase in baseline and average 25(OH)D s.c. of 1 ng/ml was associated with a significantly reduced s.c. of LDH (**Table 3**; -3.619 U/l—baseline, *p* = 0.133; -5.98 U/l—during the complete OP, *p* = 0.016; respectively) and S100P (**Table 3**; -0.038 µg/l—baseline, *p* = 0.114; -0.058 µg/l—during the complete OP, *p* = 0.075, respectively).

### Serum 25(OH)D Concentrations Are Associated With Frequency of Adverse Events of Melanoma Patients Treated With BRAF/MEK and/or Immune Checkpoint Inhibitors

**Figure 5:**
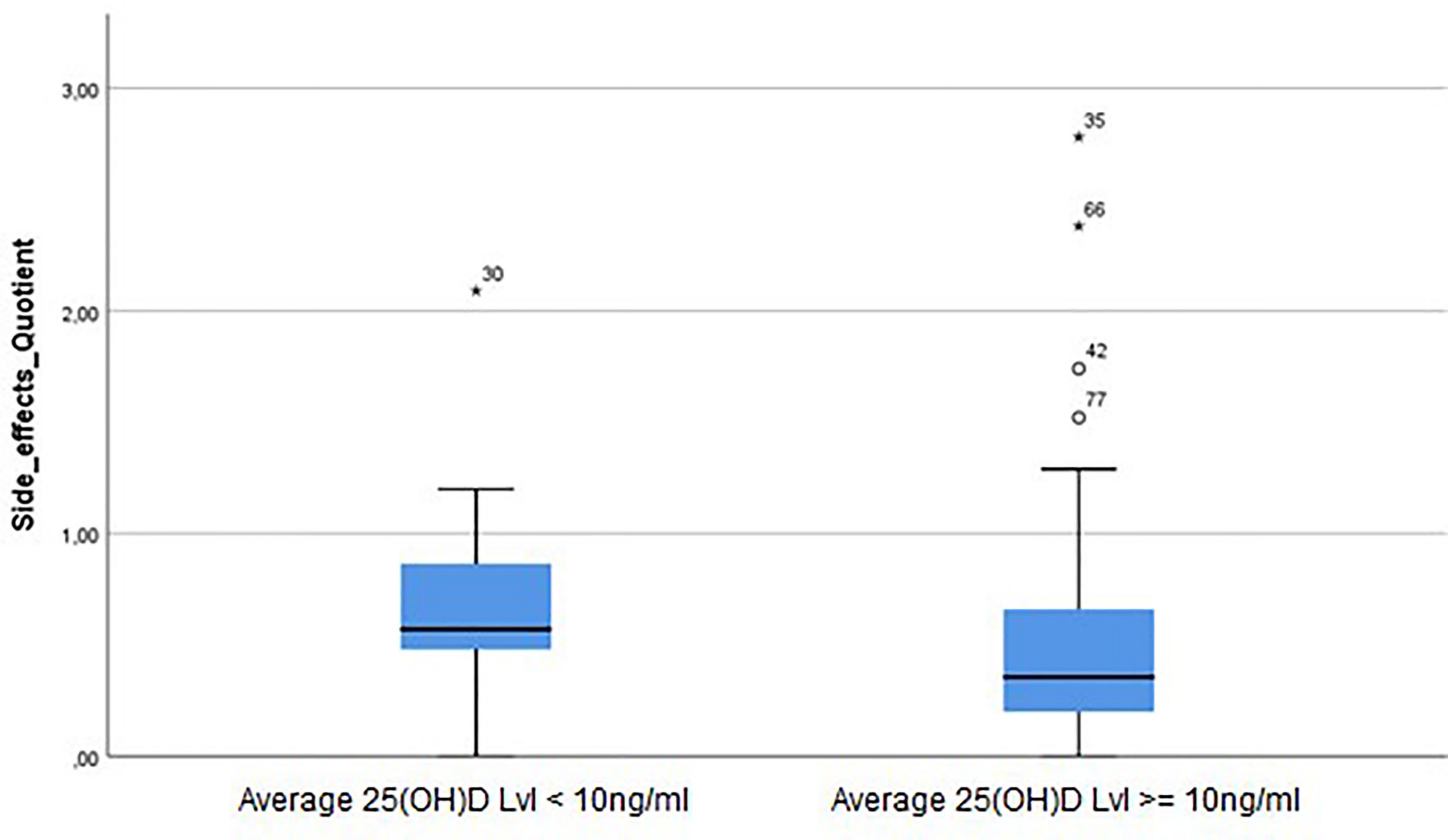
25(OH)D s.c. are associated with frequency of adverse events (AEs) in patients treated for advanced melanoma with BRAF, MEK, and/or immune checkpoint inhibitors. Analyzing the complete cohort of melanoma patients included in this study, vitamin D status was not associated with a significantly altered frequency of AEs (estimated as AE ratio that was calculated as described in the *Methods*). However, there was a trend for reduced frequency of AEs (AE ratio -0.18) in patients with average 25(OH)D serum concentrations ≥10 ng/ml as compared to severely vitamin D deficient individuals [average 25(OH)D serum concentration <10 ng/ml] (*p* = 0.303).

Analyzing all individuals of our cohort of melanoma patients included in this study, vitamin D status was not associated with a significantly altered frequency of AEs (estimated as AE ratio that was calculated as described in *Methods*) (**Figure 5**).

However, there was a trend for reduced AEs ratio (**Table 3**, AE ratio -0.180) in patients with average 25(OH)D s.c. ≥10 ng/ml as compared to severely vitamin D-deficient individuals [average 25(OH)D s.c. <10 ng/ml] (*p* = 0.303).

Moreover, an increase in average 25(OH)D s.c. of 1 ng/ml was associated with a trend for a reduced AEs ratio (**Table 3**; -0.005; *p* = 0.295). Subgroup analysis revealed that patients with *BRAF*mut melanomas and average 25(OH)D s.c. ≥10 ng/ml showed over the complete OP a trend for increased AEs ratio (**Table 3**; 0.326, p=0.289 reduced AEs ratio) as compared to severely vitamin D-deficient individuals [average 25(OH)D s.c. <10 ng/ml]. In patients with *BRAF*mut melanomas, an increase in average 25(OH)D s.c. of 1 ng/ml was associated with a non-significant increase of AEs ratio (**Table 3**; AE-ratio 0.001., p=0.765).

Patients with average 25(OH)D s.c. ≥10 ng/ml and with *BRAF*wt melanomas showed a trend for a lower AEs ratio (**Table 3**; AE ratio -0.18) as compared to severely vitamin D-deficient individuals (*p* = 0.358). In patients with *BRAF*wt melanomas, an increase in average 25(OH)D s.c. of 1 ng/ml was associated with a non-significant decrease of AEs ratio (**Table 3**; AE-ratio -0.012, *p* = 0.137).

## Discussion

We show in this comparative, non-interventional, side-by-side analysis that low vitamin D status is a strong predictor of poor outcome in patients treated for advanced melanoma with BRAFi, MEKi, CTLA-4i, and/or PD-1i. Investigating both “baseline” and “average” 25(OH)D s.c., OS was markedly reduced in severely vitamin D-deficient individuals as compared to patients with 25(OH)D s.c. ≥10 ng/ml.

Results of our study are well in line with other investigations that support the concept that vitamin D deficiency may favor poor outcome in cancer patients.

Recently, it has been shown that vitamin D regulates *via* different pathways and independent mechanism functions of many cell types involved in immunologic reactions and in the antitumor response (17–27). It was demonstrated that vitamin D is not exclusively activated through consecutive hydroxylations by cytochrome P450 family enzymes at positions C25 (by CYP2R1 or CYP27A1) and C1 (by CYP27B1) to produce 1,25(OH)_2_D_3_ but also through the action of CYP11A1 to produce mono-, di-, and trihydroxy-D_3_ products that can be further modified by CYP27B1, CYP27A1, and CYP24A1 (17–27). It has been shown that these alternative hydroxyderivatives are circulating in serum (26, 27). The active forms of vitamin D_3_, in addition to regulating calcium metabolism, exert pleiotropic activities, which include anticarcinogenic and anti-melanoma effects in experimental models, with photoprotection against UVB-induced damage (17–27). These diverse effects are mediated through an interaction with the VDR and/or as most recently demonstrated through action on retinoic acid orphan receptors (ROR)α and RORγ (21). With respect to melanoma, low levels of 25(OH)D are associated with thicker tumors and reduced patient survival (7, 8, 17, 18). Furthermore, single-nucleotide polymorphisms (SNPs) of *VDR* and the vitamin D-binding protein (*VDP*) genes affect melanomagenesis or disease outcome (17–27). Clinicopathological analyses have shown positive correlation between low or undetectable expression of VDR (20, 21) and/or CYP27B1 (22) in melanoma with tumor progression and shorter OS and disease-free survival (DFS). Paradoxically, this correlation was reversed for CYP24A1 (inactivating 24-hydroxylase) (18), indicating that this enzyme, while inactivating 1,25(OH)_2_D_3_, can activate other forms of vitamin D_3_ that are products of the non-canonical pathway initiated by CYP11A1 (25). An inverse correlation has been found between the levels of RORα and RORγ expression and melanoma progression and disease outcome (21). It has been proposed that defects in vitamin D signaling including vitamin D_3_ activation/inactivation, and the expression and activity of the corresponding receptors, affect melanoma progression and the outcome of the disease (17). Consequently, it has been postulated that the existence of multiple bioactive forms of vitamin D_3_ and alternative receptors affecting the behavior of melanoma should be taken into consideration when applying vitamin D management for melanoma therapy. In this context, it would also be interesting to know whether expression levels of VDR and/or of other proteins involved in mediating vitamin D-induced cellular signaling, or SNPs in their corresponding genes, may have an impact on the clinical outcome in patients with advanced melanoma.

Vitamin D deficiency-induced alterations in immune function, that may be of relevance for melanoma progression, include the upregulation of PDL-1 expression on both epithelial and immune cells. This finding indicates a synergic therapeutic effect in combination with ICIs, that is well in line with results of this study but needs to be further investigated. Vitamin D deficiency-induced alterations in immune function include impaired function of tumor-associated macrophages (TAMs) that may result in reduced efficacy of antitumor therapy. Infiltration by macrophages represents a characteristic morphological hallmark in many malignancies. In theory, macrophages and other immune cells involved in immune surveillance of solid tumors are able to kill malignant cells. However, melanoma cells (and many other cancer cells) are capable of escaping from immunosurveillance, and for reasons that are not yet well understood, TAMs in general do not kill them. Although macrophages can, in principle, target neoplastic cells and mediate antibody-dependent cellular cytotoxicity (ADCC), TAMs regularly fail to exert direct cytotoxic functions. Until today, the underlying mechanisms responsible for this observation remain unclear. Interestingly, Bruns et al. (9) demonstrated that inflammatory M1 macrophages kill proliferating high-grade B-cell lymphoma cells by releasing the antimicrobial peptide cathelicidin in a vitamin D-dependent fashion. They further showed that cathelicidin directly induces cell death by targeting mitochondria of these malignant cells. In contrast, anti-inflammatory M2 macrophages and M2-like TAMs exhibit an altered vitamin D metabolism, resulting in a reduced production of cathelicidin and consequently in inability to lyse malignant cells. In that study, treatment of M2 macrophages with the bioactive form of vitamin D, 1,25(OH)_2_D_3_, or a VDR agonist effectively induced cathelicidin production and triggered tumoricidal activity. Furthermore, rituximab-mediated cytotoxicity of vitamin D-treated M2 macrophages was cathelicidin-dependent. Finally, vitamin D treatment of 25(OH)D-deficient volunteers *in vivo* or primary TAMs *in vitro* improved in that study rituximab-mediated ADCC against B-cell lymphoma cells. These data indicate that activation of the vitamin D signaling pathway stimulates the antitumor activity of TAMs and improves the efficacy of ADCC. In summary, Bruns et al. (9) showed that vitamin D can help promote the antitumor activity of macrophages and stimulate their production of cathelicidin, an antimicrobial peptide that can also induce tumor cell death. The results suggest that for melanoma and other cancer patients who are deficient in vitamin D, providing vitamin D supplementation may be helpful in battling the disease and promoting the efficacy of antitumor therapy. It can be speculated that the poor outcome that we here report in patients treated for advanced melanoma with combination therapy with BRAFi and MEKi and/or with CTLA-4i or PD-1i immunotherapy may be at least in part mediated by vitamin D deficiency-induced impairment of immune function.

However, it has to be noted that the associations that we show in this observational study are no definite proof of a causal relationship. Dissociating the effects of overall fitness on responses to treatment vs. the effects of vitamin D status *per se is* difficult. In this context, we cannot exclude reverse causality, indicating that 25(OH)D s.c. at least in part might be a consequence of disease progression rather than causal.

It cannot be excluded that the association of vitamin D status with clinical outcome that we report in this study may at least in part be caused by other factors (e.g., “good health,” resulting in frequent outdoor activities) for which vitamin D status may serve as a marker for. It is well known that vitamin D status is positively associated with factors may have in turn a potential effect on melanoma survival. However, it has to be noted that when potentially interfering factors, including location of metastases (e.g., in liver or kidneys, representing organs of high importance for the vitamin D endocrine system), sex, age, history of other malignancies, diabetes mellitus, hyperlipidemia, hypercholesterinemia, coronary heart disease, heart insufficiency, heart rhythm disorders, hypertension, kidney, thyroid or liver disease, asthma bronchiale, ethanol and nicotine abuse, were assessed to adjust Cox regression modeling, none of them were identified as confounders (**Supplementary Table S1**).

Other limitations of our study include the small study size. Although case numbers in some of our analyses, including subgroup analyses, were low, subgroup analyses and results after adjusting for various parameters, including *BRAF*/*N-RAS*mut (HR: 0.138, *p* = 0.000004) and markers of TL such as average LDH (HR 0.306, *p* = 0.026) and S100P (HR 0.225, *p* = 0.003), indicate that the relationship between vitamin D status and clinical outcome in melanoma patients treated for metastasized disease with BRAFi/MEKi and/or immunotherapy may at least in part be causal. However, one has to keep in mind that the small sample size of our observational study makes it difficult to draw definite conclusions and also makes it difficult to interpret the results of subgroup analyses, e.g., looking at *BRAF*mut vs. *BRAF*wt melanomas.

Driver mutations in *BRAF* are found in approximately 50% of melanomas, play an important role in cell proliferation and metastases, and also increase inflammation by promoting secretion of pro-inflammatory cytokines, including interleukin (IL)‐6 and IL‐8 (5). Inflammation aids proliferation, survival of cancer cells, angiogenesis, and metastasis. However, there are limited data that analyze the relationship between *BRAF* and vitamin D status in patients with melanoma.

A retrospective study determined levels of serum 25(OH)D in patients with primary and metastatic melanoma and made comparisons according to age, sex, stage, season, and *BRAF* status (5). In that study, *BRAF* status was established in 93 patients, 59 of whom (63%) were positive for *BRAF* mutation. Of these, 22 patients (37%) were severely vitamin D-deficient compared with three (9%) *BRAF* wild‐type (*P* = 0.006). Of the 43 patients who died, 37 (86%) were vitamin D-deficient; 14 of the 43 deceased patients (33%) were severely vitamin D-deficient and 11 of these 14 patients (79%) were positive for *BRAF* mutation.

Studies have demonstrated a 3-fold increased risk of death for higher‐risk tumors harboring *NRAS* or *BRAF* mutations compared with wild‐type melanomas, after adjusting for other prognostic factors (28). Further studies are required to investigate the association of severe vitamin D deficiency with *BRAF*‐mutated melanoma, especially as low vitamin D levels may impact and increase the incidence of immune‐related adverse events in patients receiving immunotherapy (29). In this study, over 80% of patients with melanoma were vitamin D deficient; therefore, we recommend testing for all patients with melanoma regardless of stage.

Here we report that low vitamin D status is a strong predictor of poor outcome in patients treated for advanced melanoma with BRAFi, MEKi, CTLA-4i, or PD-1i, whether or not the tumors harbor mutations in the *BRAF* gene. However, vitamin D status-induced effects on outcome appear to be pronounced in individuals with *BRAF* mutant melanomas. In the subgroup of patients whose melanomas do or do not harbor mutations in the *BRAF* gene, individuals with average 25(OH)D serum concentrations ≥10 ng/ml had over the complete OP a 72.5% (*p* = 0.63) and 85.2% (*p* = 0.000134), respectively, reduced risk to die, as compared to individuals with average 25(OH)D serum concentrations <10 ng/ml.

Immunomodulating activities of vitamin D compounds include inhibition of T-helper lymphocytes that are often involved in the pathogenesis of immune-related adverse events (iAEs) that complicate ICI treatment, upregulation of PDL-1 expression on both epithelial and immune cells, suggesting a synergic effect in combination with ICIs, for which further investigation is needed.

Although the results were not statistically significant, our study indicates that the vitamin D status-associated improved outcome in our patients may be associated with a decreased frequency of AEs. During the complete OP, a trend for slightly reduced AEs (AE ratio -1.01) was documented in patients with average 25(OH)D serum concentrations ≥10 ng/ml as compared to severely vitamin D-deficient individuals [average 25(OH)D serum concentration <10 ng/ml] (*p* = 0.449). If this trend will be confirmed in future investigations, it can be speculated whether this decrease in the frequency of AEs may be caused by effects of vitamin D compounds on melanoma cells, immune cells, or on other cell types.

Interestingly, results of our study indicate that patients with *BRAF*mut melanomas experience more AEs than patients with *BRAF*wt melanomas. In our study, patients with *BRAF*mut melanomas and with average 25(OH)D s.c. ≥10 ng/ml showed a trend for a higher frequency of AEs, (0.326, p=0.289) as compared to severely vitamin D-deficient individuals [mean 25(OH)D s.c. <10 ng/ml]. In patients with *BRAF*mut melanomas, an increase in average 25(OH)D s.c. of 1 ng/ml was associated with an increase (trend) of the frequency of AEs (AE ratio +0.432, 0.001, p=0.765 *p* = 0.266). In contrast, patients with average 25(OH)D s.c. ≥10 ng/ml and with *BRAF*wt melanomas showed a trend toward a lower frequency of AEs as compared to severely vitamin D- deficient individuals. In patients with *BRAF* wt melanomas, an increase in average 25(OH)D s.c. of 1 0.001, p=0,765 ng/ml was associated with a non-significant decrease of the AE-ratio (-0.012, *p* = 0.137). It can be speculated whether these findings may—directly or indirectly—be caused by BRAF-induced effects on cancer and immune cells that are of importance for the therapeutic efficacy in melanoma.

These immune-modulating activities include inhibition of T-helper lymphocytes, notoriously involved in the pathogenesis of immune-related AEs (iAEs) that complicate ICI treatment, upregulation of PDL-1 expression on both epithelial and immune cells, suggesting a synergic effect in combination with ICIs, for which further investigation is needed. In this context, other factors including survivor bias that is caused by the fact that the longer people survive, the longer they have time to develop toxicities also need to be considered.

In the UK and some other countries, analysis of serum 25(OH)D concentration is recommended by the National Institute of Clinical Excellence for any new patient diagnosed with melanoma (30) but not for those with already established or metastatic disease. Based on the data presented, we would suggest to measure Vitamin D and at least avoid clear deficiency by appropriate substitution.

Further studies are warranted to elucidate the intricacies of a potential direct molecular interference of the vitamin D pathway and BRAF- or ICI-modulated pathways and intercellular signaling. A causal role of vitamin D in better outcome will only be answered by interventional studies like our group has previously pioneered in the treatment of high-grade lymphomas (29).

## Author’s Note

To take part in the Resource Identification Initiative, we used the corresponding catalog number and RRID in our article.

## Data Availability Statement

The original contributions presented in the study are included in the article/**Supplementary Material**. Further inquiries can be directed to the corresponding author.

## Ethics Statement

The studies involving human participants were reviewed and approved by Ethik-Kommission der Ärztekammer des Saarlandes Faktoreistr.4 66111 Saarbrücken, Germany. Written informed consent for participation was not required for this study in accordance with the national legislation and the institutional requirements.

## Author Contributions

Substantial contributions to conception and design: JR, FB, and JS. Acquisition of data and/or analysis and interpretation of results: JR, FB, JS, SW, RS, and CP. Substantial scientific and intellectual contributions to the drafting or rewriting of the initial and/or revised article: JR, FB, JS, SW, TV, RS, and CP. Approval of the final accepted version of the article: JR, FB, SW, JS, CP, RS, and TV.

## Conflict of Interest

Saarland University, with JR and TV as principal investigators, received funding from the Jörg Wolff Foundation (Stuttgart, Germany). JR received speaker’s honoraria from Leo Pharma (Neu-Isenburg, Germany) and Cogitando (Neunkirchen, Germany).

The remaining authors declare that the research was conducted in the absence of any commercial or financial relationships that could be construed as a potential conflict of interest.

## Publisher’s Note

All claims expressed in this article are solely those of the authors and do not necessarily represent those of their affiliated organizations, or those of the publisher, the editors and the reviewers. Any product that may be evaluated in this article, or claim that may be made by its manufacturer, is not guaranteed or endorsed by the publisher.
