# Supplementary material for: Low Vitamin D Status Predicts Poor Clinical Outcome in Advanced Melanoma Treated With Immune Checkpoint or BRAF/MEK Inhibitors: A Prospective Non-Interventional Side-by-Side Analysis
Source: Front Oncol. 2022 May 20;12:839816. doi: 10.3389/fonc.2022.839816 (PMC9166268; doi:10.3389/fonc.2022.839816)
Supplement: Supplementary file 1 [file DataSheet_1.docx]

**S1 Fig: Association of OS with baseline 25(OH)D s.c.: analysis of subgroup of patients with *BRAF*mut melanomas treated for advanced disease with ICIs and/or BRAF/MEK inhibitors.**

In patients with *BRAF*mut melanomas, OS was reduced in the subgroup of patients with baseline 25(OH)D s.c. < 10 ng/ml (mean OS 51.0 weeks) as compared to the subgroup of patients with baseline 25(OH)D s.c. >/= 10 ng/ml (mean OS 105.86 weeks), respectively (*p*=0.173). After one year, 50.0% and 71.3%, after two years 16.7% and 51.0%, after three years 0% and 25% and after 4 years 0% and 25% were alive, in the subgroups of patients with *BRAF*mut melanomas and with baseline 25(OH)D s.c. < 10 ng/ml and >/= 10 ng/ml, respectively (*p*=0.173). Rounding error may occur. Number of patients may differ from total sample sizes due to missing data.


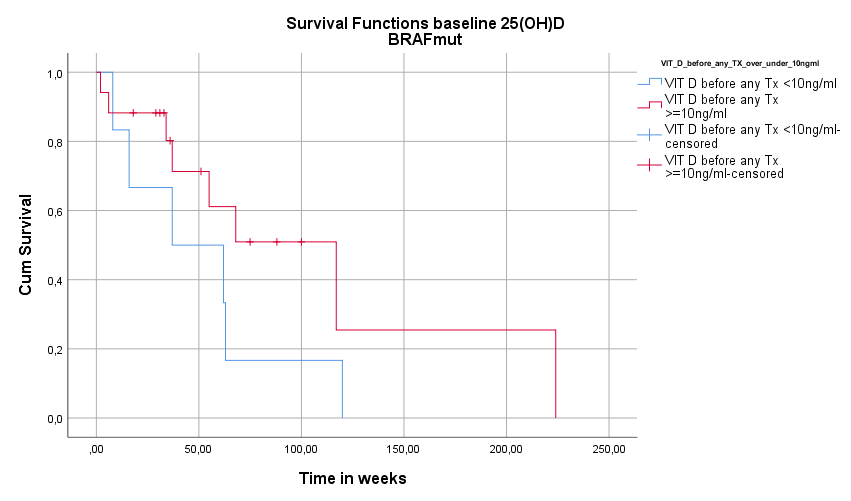


**S2 Fig: Association of OS with average 25(OH)D s.c.: analysis of subgroup of patients with *BRAF*mut melanomas treated for advanced disease with immune checkpoint inhibitors (ICIs) and/or BRAF/MEK inhibitors.**

In patients with *BRAF*mut melanomas, OS was reduced in the subgroup of patients with average 25(OH)D s.c. < 10 ng/ml (mean OS 29.0 weeks) as compared to the subgroup of patients with average 25(OH)D s.c. >/= 10 ng/ml (mean OS 93.4 weeks), respectively (*p*=0.045). After one year, 33.3% and 69.6%, after two years 0% and 46.4%, after three years 0% and 15.5% and after four years 0% and 15.5% of individuals were alive in the subgroups of patients with average 25(OH)D s.c. < 10 ng/ml and >/= 10 ng/ml, respectively (*p*=0.045). Rounding error may occur. Number of patients may differ from total sample sizes due to missing data.


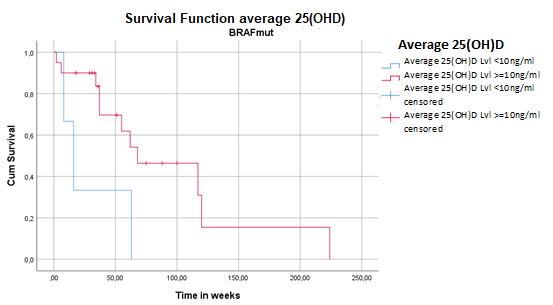


**S3 Fig: Association of Hazard function of death with baseline 25(OH)D s.c.: analysis of subgroup of patients with *BRAF*mut melanomas treated for advanced disease with ICIs and/or BRAF/MEK inhibitors.**

Patients with *BRAF*mut melanomas with baseline 25(OH)D s.c. >/= 10 ng/ml showed a trend for a reduced risk to die as compared with severely vitamin D deficient patients (HR 0.473, *p*=0.185). For patients with BRAF mutant melanomas, risk to die was during the complete OP reduced by 52.7% in individuals with baseline 25(OH)D serum concentration >/= 10 ng/ml as compared with severely vitamin D deficient patients (*p*=0.185). Rounding error may occur. Number of patients may differ from total sample sizes due to missing data.


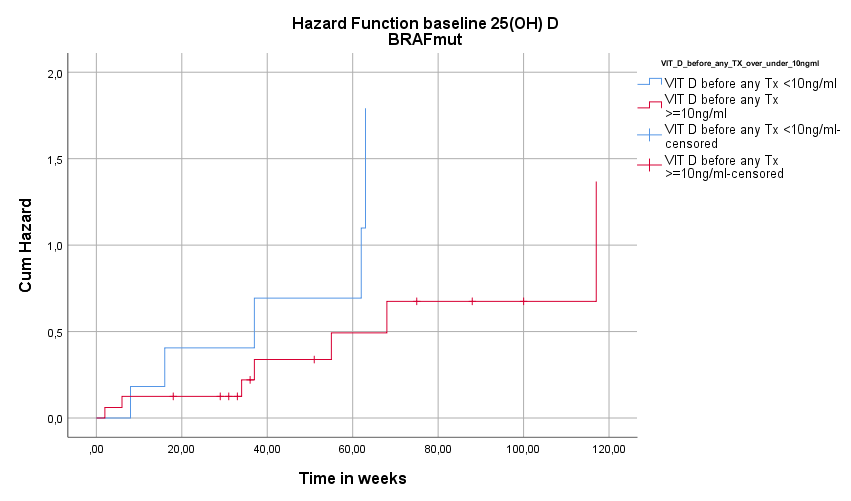


**S4 Fig: Association of Hazard function of death with average 25(OH)D s.c.: analysis of subgroup of patients with *BRAF*mut melanomas treated for advanced disease with ICIs and/or BRAF/MEK inhibitors.**  Patients with *BRAF*mut melanomas with average 25(OH)D s.c. >/= 10 ng/ml showed a trend for a reduced risk to die as compared with severely vitamin D deficient patients (HR 0.275, *p*=0.063). Risk to die was during the complete OP reduced by 72.5% in individuals with average 25(OH)D s.c. >/= 10 ng/ml as compared with severely vitamin D deficient patients. Rounding error may occur. Number of patients may differ from total sample sizes due to missing data.


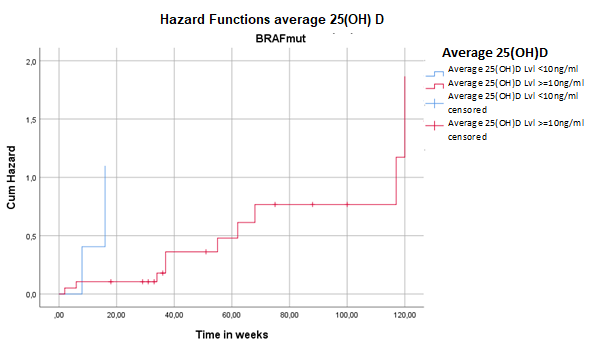


**S5 Fig: Association of OS with baseline 25(OH)D s.c.: analysis of subgroup of patients with *BRAF*wt melanomas treated for advanced disease with ICIs and/or BRAF/MEK inhibitors.**

In patients with *BRAF*wt melanomas, OS was reduced in the subgroup of patients with baseline 25(OH)D s.c. < 10 ng/ml (mean OS 81.14 weeks) as compared to the subgroup of patients with baseline 25(OH)D s.c. >/= 10 ng/ml (mean OS 118.45 weeks), respectively (*p*=0.046). OS In the subgroup of patients with *BRAF*wt melanomas, after one year, 51.0% and 70.9%, after two years 51.0% and 43.9%, after three years 0% and 43.9% and after 4 years 0% and 14.6% of individuals with baseline 25(OH)D s.c. < 10 ng/ml and >/= 10 ng/ml, respectively, were alive (*p*=0.046). Rounding error may occur. Number of patients may differ from total sample sizes due to missing data.

.


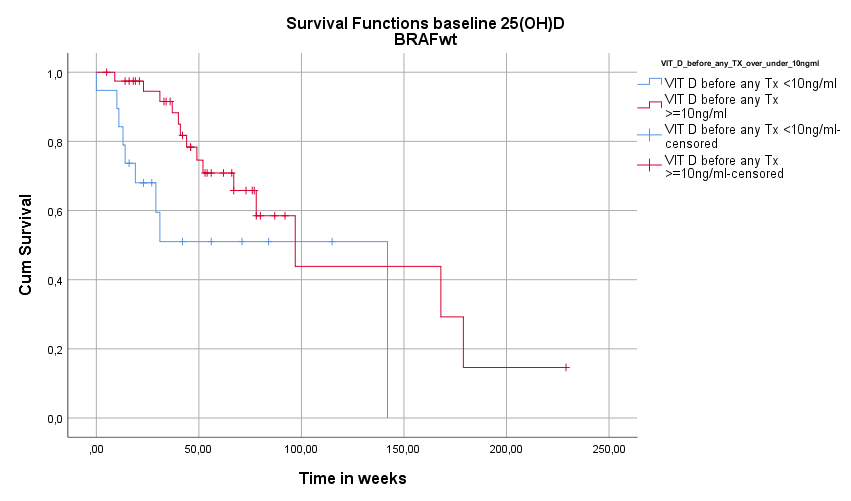


**S6 Fig: Association of OS with average 25(OH)D s.c.: analysis of subgroup of patients with *BRAF*wt melanomas treated for advanced disease with ICIs and/or BRAF/MEK inhibitors.**

In patients with *BRAF*wt melanomas, OS was reduced in the subgroup of patients with average 25(OH)D s.c. < 10 ng/ml (mean OS 29.92 weeks) as compared to the subgroup of patients with average 25(OH)D s.c. >/= 10 ng/ml (mean OS 121.53 weeks), respectively (*p*=0.000012). In patients with *BRAF*wt melanomas, after one year, 24.0% and 73.1%, after two years 0% and 52.5%, after three years 0% and 39.4% and after 4 years 0% and 13.1% of individuals were alive in the subgroups of patients with average 25(OH)D s.c. < 10 ng/ml and >/= 10 ng/ml, respectively *(p*=0.000012). Rounding error may occur. Number of patients may differ from total sample sizes due to missing data.


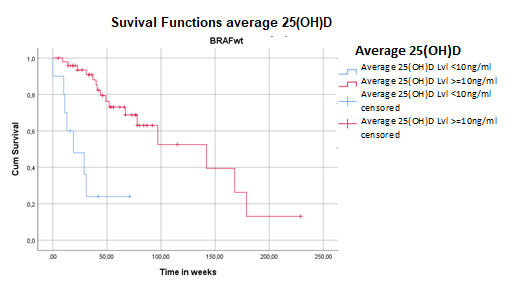


**S7 Fig:**  **Association of Hazard function of death with baseline 25(OH)D s.c.: analysis of subgroup of patients with *BRAF*wt melanomas treated for advanced disease with ICIs and/or BRAF/MEK inhibitors.**

Patients with *BRAF*wt melanomas with baseline 25(OH)D s.c. >/= 10 ng/ml showed a trend for a reduced risk to die as compared with severely vitamin D deficient patients (HR 0.421, *p*=0.053). In the subgroup of patients with *BRAF*wt melanomas, individuals with baseline 25(OH)D s.c. >/= 10 ng/ml had over the complete OP a trend for a by 57.9% reduced risk to die, as compared to individuals with baseline 25(OH)D s.c. < 10 ng/ml (HR 0.421, *p*=0.053). Rounding error may occur. Number of patients may differ from total sample sizes due to missing data.


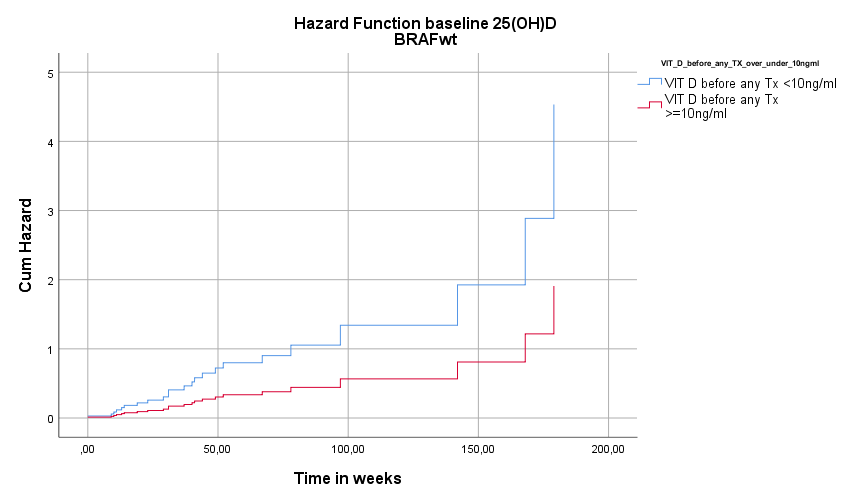


**S8 Fig: Association of Hazard function of death with average 25(OH)D s.c.: analysis of subgroup of patients with *BRAF*wt melanomas treated for advanced disease with ICIs and/or BRAF/MEK inhibitors.**

Patients with *BRAF*wt melanomas with average 25(OH)D s.c. >/= 10 ng/ml showed a trend for a reduced risk to die as compared with severely vitamin D deficient patients (HR 0.148, *p*=0.000134). Patients with *BRAF*wt melanomas, risk to die was during the complete observation period (228 weeks/mean 54.31 weeks) reduced by 85.2% in individuals with average 25(OH)D s.c. >/= 10 ng/ml as compared with severely vitamin D deficient patients (*p*=0.000134). Rounding error may occur. Number of patients may differ from total sample sizes due to missing data.


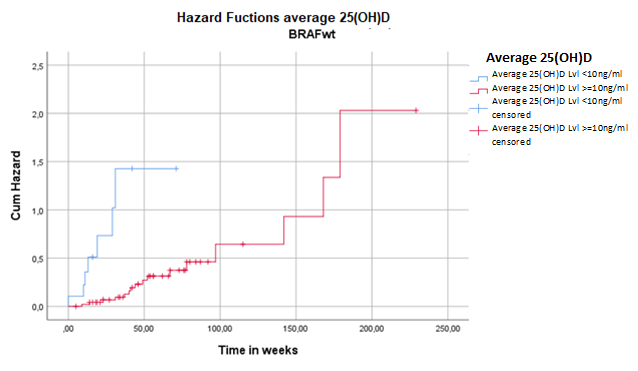


**S9 Fig: Association of Hazard function of progress with average 25(OH)D s.c.: analysis of subgroup in patients with *BRAF* mutant (mut) melanomas treated with ICIs and/or BRAF/MEK inhibitors.**

In patients with *BRAF*mut melanomas and with average 25(OH)D s.c. >/= 10 ng/ml, the risk for a progress of the disease was not significantly by 43.5% reduced, as compared to vitamin D severely deficient individuals (average 25(OH)D s.c. < 10 ng/ml) (*p*=0.479). Rounding error may occur. Number of patients may differ from total sample sizes due to missing data.

**
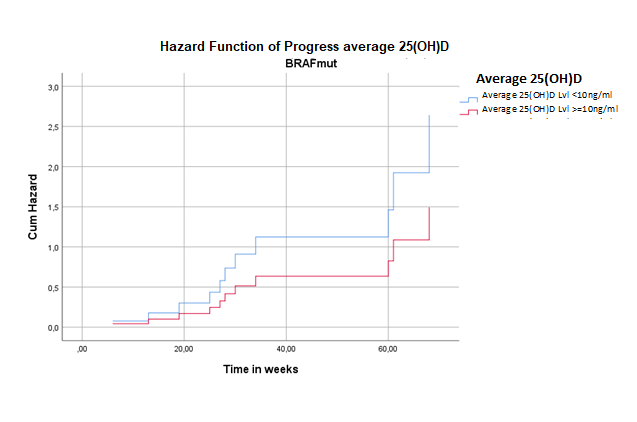
**

**Supplementary file 10/supplementary table I:**

Cox analysis of risk to die (HR) of melanoma patients after adjusting for potential confounders

**__________________________________________________________________________________________________________________**

*Average 25(OH)D s.c.* **HR 95% CI *p*-value (n=)**

**__________________________________________________________________________________________________________________**

*All* patients < 10 ng/ml vs >/= 10 ng/ml 0.179 0.08 – 0. 39 0.000016 82

__________________________________________________________________________________________________________________

*Adjusted for:*

Gender < 10 ng/ml vs >/= 10 ng/ml 0.181 0.08 – 0.40 0.000020 82

Age < 10 ng/ml vs >/= 10 ng/ml 0.176 0.08 – 0.39 0.000015 82

Ethanol abusus < 10 ng/ml vs >/= 10 ng/ml 0.180 0.08 - 0.39 0.000018 82

Nicotine abusus < 10 ng/ml vs >/= 10 ng/ml 0.173 0.08 – 0.38 0.000016 82

COPD < 10 ng/ml vs >/= 10 ng/ml 0.183 0.08 – 0.40 0.000022 82

Asthma bronchiale < 10 ng/ml vs >/= 10 ng/ml 0.179 0.08 – 0.39 0.000017 82

Diabetes mellitus (type I & II) < 10 ng/ml vs >/= 10 ng/ml 0.179 0.08 – 0.4 0.000016 82

Arterial hypertension < 10 ng/ml vs >/= 10 ng/ml 0.178 0.08 – 0.39 0.000016 82

Coronary heart disease < 10 ng/ml vs >/= 10 ng/ml 0.179 0.08 - 0.39 0.000017 82

Heart insuffiency < 10 ng/ml vs >/= 10 ng/ml 0.179 0.08 – 0.36 0.000019 82

Heart rhythm disorders * < 10 ng/ml vs >/= 10 ng/ml 0.155 0.7 – 0.34 0.0004 82

History of kidney disease < 10 ng/ml vs >/= 10 ng/ml 0.175 0.08 – 0.381 0.000012 82

History of liver disease < 10 ng/ml vs >/= 10 ng/ml 0.177 0.08 – 0.39 0.000017 82

History of thyroid disease < 10 ng/ml vs >/= 10 ng/ml 0.187 0.84 – 0.41 0.000033 82

Lipid- and cholesterol- hyperaemia < 10 ng/ml vs >/= 10 ng/ml 0.174 0.79 – 0.38 0.000015 82

History of other malignancies < 10 ng/ml vs >/= 10 ng/ml 0.167 0.08 – 0.37 0.000008 82

Liver metastases before therapy < 10 ng/ml vs >/= 10 ng/ml 0.179 0.08 – 0.39 0.000017 82

LIver metastases during OP < 10 ng/ml vs >/= 10 ng/ml 0.176 0.08 – 0.39 0.000016 82

Kidney metastases during OP < 10 ng/ml vs >/= 10 ng/ml 0.174 0.08 – 0.38 0.000012 82

**__________________________________________________________________________________________________________________**

**Abbreviations:** COPD: chronic obstructive pulmonary disease; n: number; OP: observation period; s.c.: serum concentration.
